# Fractal Analysis Reveals Reduced Complexity of Retinal Vessels in CADASIL

**DOI:** 10.1371/journal.pone.0019150

**Published:** 2011-04-27

**Authors:** Michele Cavallari, Teresa Falco, Marina Frontali, Silvia Romano, Francesca Bagnato, Francesco Orzi

**Affiliations:** 1 Department of Neurosciences, Mental Health and Sensory Organs, Sant'Andrea Hospital, University of Rome “La Sapienza”, Rome, Italy; 2 Institute of Neurobiology and Molecular Medicine, National Research Council, Rome, Italy; 3 Center for Experimental Neurological Therapies, Sant'Andrea Hospital, University of Rome “La Sapienza”, Rome, Italy; 4 Department of Radiology and Radiological Sciences, Vanderbilt University Institute of Imaging Science, Nashville, Tennessee, United States of America; Institute Biomedical Research August Pi Sunyer (IDIBAPS) - Hospital Clinic of Barcelona, Spain

## Abstract

The Cerebral Autosomal Dominant Arteriopathy with Subcortical Infarcts and Leukoencephalopathy (CADASIL) affects mainly small cerebral arteries and leads to disability and dementia. The relationship between clinical expression of the disease and progression of the microvessel pathology is, however, uncertain as we lack tools for imaging brain vessels *in vivo*. Ophthalmoscopy is regarded as a window into the cerebral microcirculation. In this study we carried out an ophthalmoscopic examination in subjects with CADASIL. Specifically, we performed fractal analysis of digital retinal photographs. Data are expressed as mean fractal dimension (mean-D), a parameter that reflects complexity of the retinal vessel branching. Ten subjects with genetically confirmed diagnosis of CADASIL and 10 sex and age-matched control subjects were enrolled. Fractal analysis of retinal digital images was performed by means of a computer-based program, and the data expressed as mean-D. Brain MRI lesion volume in FLAIR and T1-weighted images was assessed using MIPAV software. Paired t-test was used to disclose differences in mean-D between CADASIL and control groups. Spearman rank analysis was performed to evaluate potential associations between mean-D values and both disease duration and disease severity, the latter expressed as brain MRI lesion volumes, in the subjects with CADASIL. The results showed that mean-D value of patients (1.42±0.05; mean±SD) was lower than control (1.50±0.04; p = 0.002). Mean-D did not correlate with disease duration nor with MRI lesion volumes of the subjects with CADASIL. The findings suggest that fractal analysis is a sensitive tool to assess changes of retinal vessel branching, likely reflecting early brain microvessel alterations, in CADASIL patients.

## Introduction

The Cerebral Autosomal Dominant Arteriopathy with Subcortical Infarcts and Leucoencephalopathy (CADASIL) affects small penetrating cerebral and leptomeningeal arteries. Thickening of the arterial wall leads to lumen stenosis, microthrombi formation and ischemic lesions [1].

Knowledge on the pathophysiology of CADASIL mainly relies upon *post mortem* data. Due to lack of methods to inspect brain microvessels *in vivo*, early pathogenic mechanisms of the disease remain elusive. Cerebral microbleeds and dilated Virchow–Robin spaces represent early tissue changes, which can be detected by magnetic resonance imaging (MRI). Animal models suggest that these changes represent the result of long-lasting dysfunctions within the neurovascular unit [2] and precede white matter (WM) lesion formation [3]. It remains, however, unproven at which stage the small vessel alterations occur in humans. Questions also remain on the relation between the extent of vessel damage and WM lesion volume. In order to address this issue we carried out an ophthalmoscopic examination in subjects with CADASIL. Ophthalmoscopic examination is, in fact, regarded as a window into the cerebral microcirculation [4], as retinal microvascular abnormalities are predictive of lacunar stroke [5], high stroke incidence [6], WM lesions [7], cognitive impairment [8] and CADASIL [9].

Specifically, we performed fractal analysis of the retinal vessels. Fractal analysis allows us to obtain a measure of complexity, or density, of the retinal vessel branching [10], which is expressed by the mean fractal dimension (mean-D) value.

We found that mean-D value was lower in CADASIL than control subjects, suggesting that fractal analysis is a sensitive tool for quantitatively detecting microvessel changes.

## Methods

Written informed consent was obtained from all participants involved in the study; Sant'Andrea Hospital Ethics Committee approved the study.

Ten patients with CADASIL and 10 age- (±3 years) and sex-matched healthy volunteers were enrolled. Patients from 5 families of central and southern Italy, diagnosed with CADASIL by molecular diagnosis, were consecutively enrolled. Four subjects were asymptomatic and were evaluated because of familiarity. Clinical history was first collected from each subject to rule out other diseases and to record time of disease onset, clinical features and risks factors for cerebrovascular accidents, such as diabetes, hypertension and smoking. Each person underwent a physical exam and ophthalmoscopy. Within three months from the ophthalmoscopic evaluation an MRI was obtained from each patient.

Molecular diagnosis was performed by sequencing the exons and intron-exon boundaries of Notch3 gene (starting from exon 3 and 4, most often carrying mutation). All patients had the common type of CADASIL mutation, i.e. missense mutations leading to the loss or gain of a cysteine residue, namely Arg90Cys (exon 3), Arg133Cys, Cys123Ser (exon 4) or Cys446Trp (exon 8).

Retinal photographs were obtained using a digital fundus camera. Digital images were processed by using ImageJ (http://rsb.info.nih.gov/ij), in order to isolate the retinal vessels. Briefly, following green-filtering of the original red-green-blue format, a circular region-of-interest of 3.5 optic disc radii, centered on the optic disc, was selected for the analysis. Background subtraction was performed using the mean optical density value from 4 arbitrarily selected, vessel-free, areas (60×60 pixels). The images were then converted into binary format, and the edge of the vessels was automatically traced, using the binary/outline function of the software ImageJ. Artifacts, mostly due to peripapillary atrophy, were identified and removed. Fractal analysis of the retinal vessels was finally carried out by means of the FracLac plug-in (http://rsb.info.nih.gov/ij/plugins/fraclac/FLHelp/Introduction.htm) according to the box-counting approach [10]. The fractal analysis is an operator-independent procedure. The result of the analysis is expressed with a single value, the mean fractal dimension (mean-D), which quantifies the complexity, or density, of the retinal vessel branching. The analysis was performed by a trained physician (MC), blind to the demographic, clinical and neuroimaging data.

MRI was performed using a 1.5-T magnet equipped with a standard head coil. Axial 2Dimensional (2D) Fluid Attenuated Inversion Recovery (FLAIR) images (echo time [TE] 150–200 ms, repetition time [TR] 6000–10000 ms) and pre-contrast T1-Weighted (T1-W) Spin Echo (SE) (TE 10-20 ms, TR 550–700 ms) images were acquired. For each sequence, full brain coverage with 3-mm thick contiguous slices with 250-mm field of view and in plane 256×256 matrix size, was obtained. The total volume of the hyperintense lesions in FLAIR, and of the hypointense lesions (or black holes) in T1-W SE images were measured, as previously described [11], [12]. As active lesions are usually not present in CADASIL patients no post-contrast sequences were acquired and it was assumed that all WM lesions in FLAIR and T1-W SE represented chronic lesions. One blind observer (TF) measured the WM lesion volume in both sequences using the Medical Image Processing, Analysis, and Visualization (MIPAV) software (http://mipav.cit.nih.gov) as previously described [11]. Subsequently, each mask was inspected by a senior investigator (FB).

Group-differences in mean-D were analyzed using paired t-tests. Spearman rank correlation analysis was employed to test association between mean-D and age, in each group separately, and in all the subjects from both groups together. Spearman rank correlation analysis was also performed in order to investigate the association between mean-D and both disease duration and disease severity, within the CADASIL patients. Disease duration was expressed as number of years from clinical onset, while disease severity was expressed as MRI lesion volume in both FLAIR and T1-W SE images. Statistical analyses were performed using the Graph Pad software (www.graphpad.com).

## Results

Table 1 reports the demographic and clinical data of the study cohort. Mean age was 43.4 ± 8 (mean ± SD) in both groups, and there was no difference between patients and controls in the prevalence of cerebrovascular risk factors (smoking was equally distributed between the groups, and major risk factors, i. e. diabetes and hypertension, were absent in both groups).

**Table 1:** Demographic and clinical data of the study cohort.

	Patients				Healthy Volunteers		
	*Age*	*Sex*	*Main clinical features (age of onset)*	*Risk Factors*	*Age*	*Sex*	*Risk Factors*
*Pair 1*	49	F	Seizures (44)	None	48	F	None
*Pair 2*	51	M	Stroke (40)	Smoker/Dyslipidemia	51	M	Previous smoker
*Pair 3*	36	F	None	None	36	F	Previous smoker
*Pair 4*	58	F	None	None	56	F	None
*Pair 5*	38	F	Migraine (35)	None	39	F	None
*Pair 6*	45	F	None	None	42	F	None
*Pair 7*	52	M	Stroke	Dyslipidemia	53	M	None
*Pair 8*	36	F	Migraine, TIAs* (33)	PFO**	35	F	None
*Pair 9*	35	M	None	Previous smoker	33	M	Smoker
*Pair 10*	38	M	Migraine (23)	Obesity	41	M	None

* TIAs  =  transient ischemic attacks.

** PFO =  patent foramen ovale.

**Figure 1:**
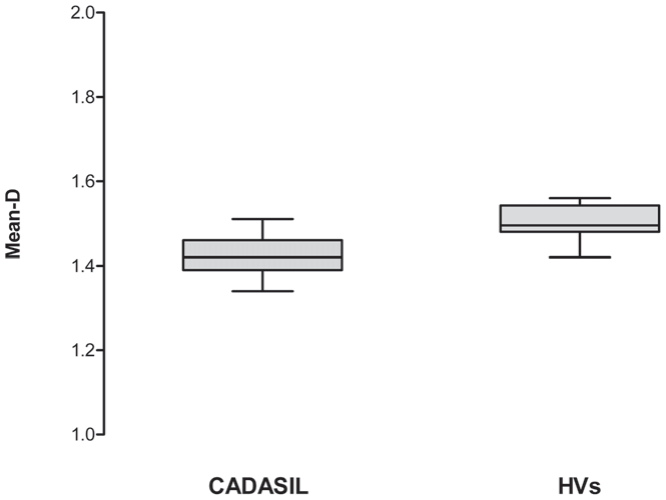
Box plot illustrating group-differences in mean fractal dimension (mean-D). The boxes include data between 25th and 75th percentiles. Horizontal line in the box represents the median value. The whiskers indicate the minimum and maximum values. (HVs  =  Heathy Volunteers)

**Figure 2:**
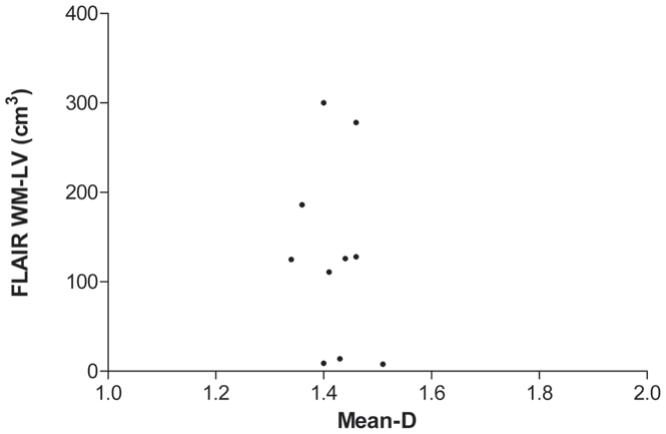
Scatter plot showing the lack of correlation between white matter lesion volume (WM-LV) in FLAIR images and fractal analysis dimension (mean-D).

**Figure 3:**
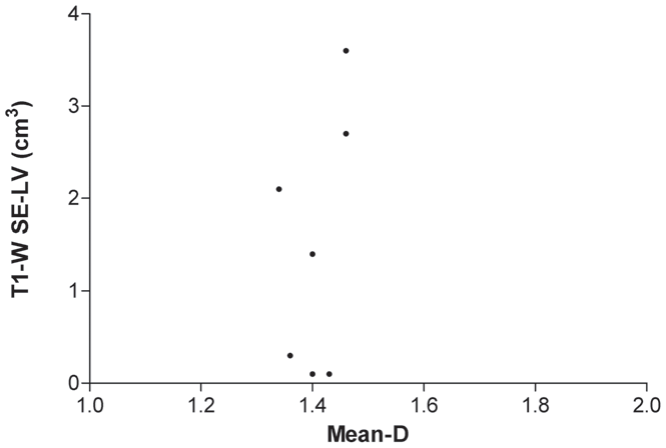
Scatter plot showing the lack of correlation between white matter lesion volume (WM-LV) in T1-W SE images and mean fractal dimension (mean-D).

Mean-D values were lower (p = 0.002) in patients compared to controls (fig. 1). There was no significant correlation between mean-D and age neither in all the subjects of both groups together (r = −0.3, p = 0.14), nor in the patient (r = −0.5, p = 0.14) or in the control (r = −0.4, p = 0.2) group. There was also no significant correlation between retinal mean-D and brain MRI lesion volume, as assessed by FLAIR (r = −0.2, p = 0.66, fig. 2), or T1-W SE imaging (r = −0.1, p = 0.73, fig. 3).

Of the ten examined patients, all had WM lesions in FLAIR images and seven had black holes in T1-W SE images. Consistently, a large WM lesion volume in FLAIR images was associated with large black holes lesion volume (r = 0.7, p = 0.04).

**Figure 4:**
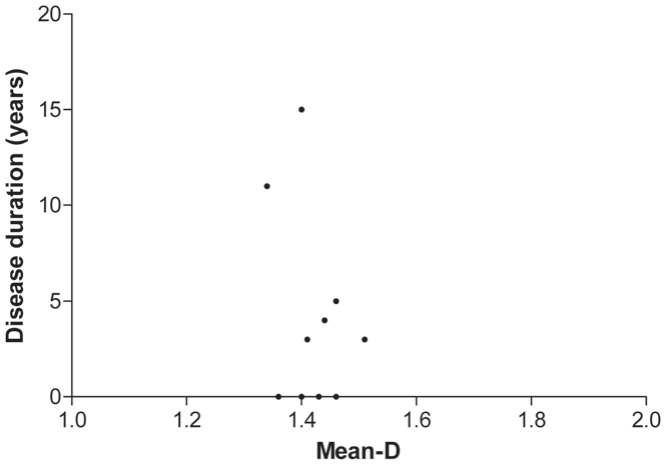
Scatter plot showing the lack of correlation between mean fractal dimension (mean-D) and disease duration, expressed as years from clinical onset.

Within CADASIL group, the duration of the disease ranged from 0 (4 subjects were asymptomatic) to 15 years. No correlation was found between mean-D and disease duration (r = −0.1, p = 0.75, fig. 4).

## Discussion

The main findings of our study are: (1) fractal analysis reveals reduced density of the retinal vessel branching in CADASIL patients, and (2) the finding does not relate to disease duration and disease severity.

It is a substantial hypothesis that the reduced mean-D in our patients mirrors the cerebral microvascular changes associated with CADASIL. Previous reports have suggested a possible role for fractal analysis of the retinal vessels as a diagnostic mean to detect systemic or cerebral vascular abnormalities. For instance, changes in mean-D have been reported in subjects with lacunar stroke [13], [14]. In one case the authors found a decrease in mean-D [13], in the other an increment [14]. The increment in mean-D was postulated to reflect increased angiogenesis following acute stroke [14]. Consistently, the reduced branching of the retinal vessels reported in our study could reflect the chronic progression of the CADASIL and the ineffectiveness of the Notch signaling in vascular remodeling [15]. Although such an interpretation of the finding remains questionable, it is reasonable to assume that the decreased fractal dimension observed in CADASIL reflects the thickening of the arterial wall, lumen stenosis, and consequent vessel occlusion that might be common both to CADASIL and lacunar or small vessel disease stroke.

Brain parenchyma damage was documented by MRI in all our patients. The majority of them also presented with black holes in T1-W SE images as well. The latter, are lesions with longer T1, shown to represent areas of advanced axonal pathology in other brain diseases [12]. Yet, no association was found between retinal fractal dimension and tissue damage, as revealed by FLAIR or T1-W MRI. Consistently there was also no association between retinal fractal dimension and disease duration.

Obviously, the small number of subjects included in the study precludes any definitive interpretation. Notwithstanding the above reservation, one can reasonably postulate that the observed retinal vascular changes represent relevant component of the disease, and that they reflect pathogenic mechanisms common to the alterations of the brain vessel and parenchyma. Retinal fractal analysis seems, therefore, to be a simple, sensitive, and operator independent tool to assess changes of retinal vessel branching, likely reflecting brain microvessel alterations, in CADASIL patients. A remaining question is to what extent changes in retinal vessel branching reflect the degree of disease in CADASIL patients. The apparent lack of association between retinal fractal dimension and both duration of the disease and extent of the brain pathology (as assessed by MRI) suggests that changes in the retinal vessels are early signs of the disease.
